# Cross-subject generalization for EEG emotion recognition: a review of methods, challenges, and future trends

**DOI:** 10.3389/fncom.2026.1865513

**Published:** 2026-07-02

**Authors:** Zhengping Li, Xiaofeng Wu, Yuwen Hao, Lijun Wang, Xiaoxue Li, Hao Duo

**Affiliations:** 1 College of Artificial Intelligence and Computer Science, North China University of Technology, Beijing, China; 2 Disaster Medicine Research Center, Medical Innovation Center of PLA General Hospital, Beijing, China; 3 Hangzhou Institute of Technology, Xidian University, Hangzhou, China; 4 China Academy of Information and Communications Technology, Beijing, China

**Keywords:** BCI, cross-subject, domain adaptation, EEG, emotion recognition, transfer learning

## Abstract

Cross-subject emotion recognition based on electroencephalogram (EEG) signals faces significant challenges, mainly because EEG data are highly non-stationary and easily influenced by time, environment, and individual physiological states. Meanwhile, substantial inter-subject variability leads to obvious differences in signal patterns across different people, which makes it difficult for a single model to learn stable and transferable emotional features. As a result, these factors severely hinder model generalization and reduce recognition performance in real-world applications. Unlike previous reviews that categorize methods based on network architectures, this paper proposes a novel taxonomy grounded in the “generalization hypothesis,” synthesizing existing approaches into five major paradigms: statistical and adversarial distribution alignment, topological and structural modeling, advanced representation learning, generative modeling and style reconstruction, and multimodal complementary fusion. Our analysis reveals that the core conflict lies in the trade-off between alignment intensity and semantic integrity. Future research should integrate causal representation learning with source-free domain adaptation to realize truly plug-and-play affective brain–computer interfaces (aBCIs).

## 1 Introduction

Emotion is a complex psychological and physiological state triggered by specific events or situational stimuli, profoundly influencing individual perception, decision-making and behavior. Therefore, accurate emotion recognition holds significant research value in fields such as human-computer interaction (HCI), mental health, and cognitive science (Waters et al., 1993). Traditional emotion recognition methods primarily rely on non-physiological signals such as facial expressions, speech intonation and body language (Kessous et al., 2010). However, these modalities are susceptible to intentional suppression or manipulation, leading to subjective limitations. In contrast, physiological signals—particularly electroencephalogram (EEG) signals—have garnered widespread attention for their ability to directly capture neural activity in the cerebral cortex, enabling more precise and reliable emotion recognition (Tawsif et al., 2022).

However, these successes rely on a critical assumption: that the training data (source domain) and the testing data (target domain) originate from the same group of individuals and satisfy the independent and identically distributed (i.i.d.) condition. As EEG-based affective brain-computer interfaces (aBCIs) transition from laboratory settings to real-world applications, models suffer from poor generalization capability under cross-subject conditions. Traditional subject-dependent models require every new user to undergo time-consuming data collection and calibration processes, which severely compromises user experience and system usability. To overcome this hurdle, cross-subject emotion recognition—which involves training a general model using data from existing users (source domain) and generalizing it to unseen users (target domain)—has become a core objective in current research.

Achieving robust cross-subject generalization is extremely difficult, primarily due to two fundamental issues: non-stationarity and inter-subject variability. Specifically, even within the same subject, the statistical properties of EEG signals drift over time. Furthermore, variations in brain anatomical structures among individuals (e.g., skull thickness, cortical folding) cause physical shifts in the spatial distribution of scalp potentials, while differences in psychological baselines and cognitive appraisal patterns lead to semantic shifts in emotional responses.

Although numerous deep learning methods have attempted to mitigate these shifts, existing reviews mostly categorize works based solely on backbone network architectures (e.g., “CNN-based methods”). This architecture-oriented perspective often obscures the underlying theoretical mechanisms of how models actually achieve generalization. To fill this gap, this paper proposes a comprehensive review and a novel taxonomy centered on generalization mechanisms.

**Figure 1:**
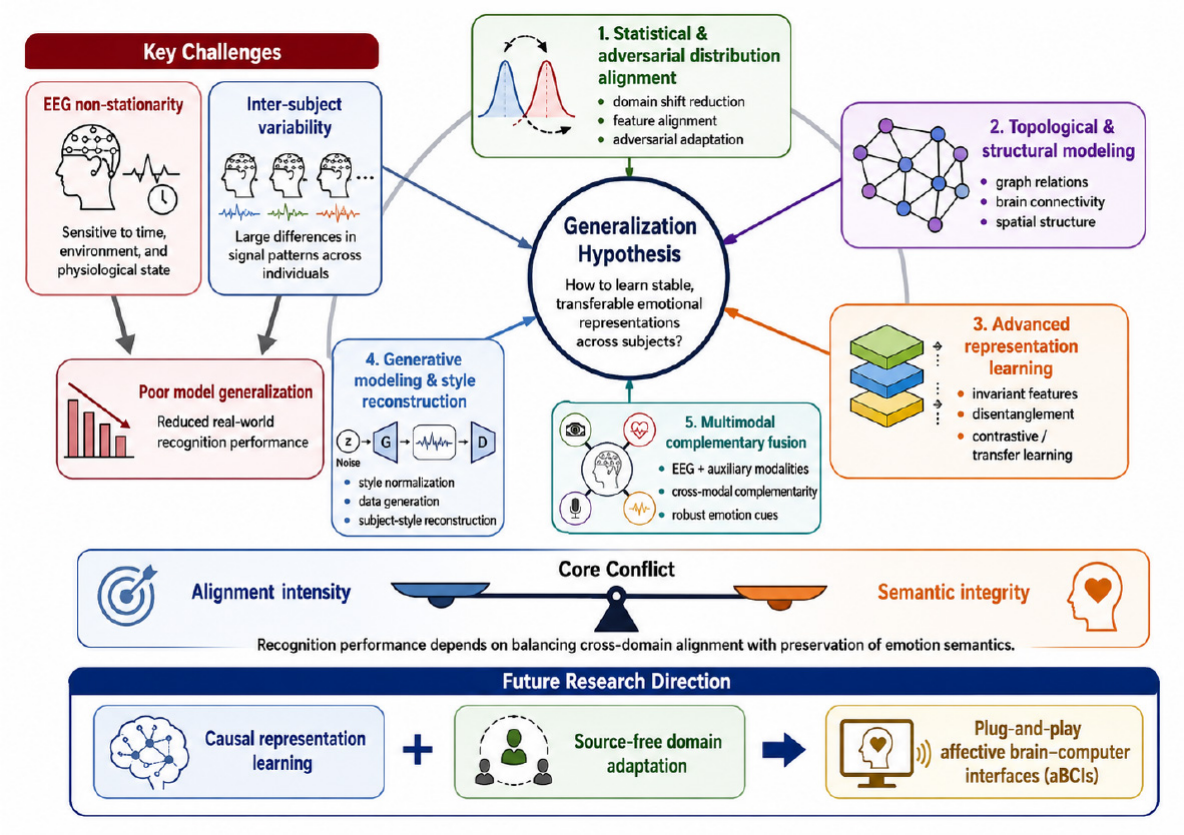
Systematic framework of the proposed taxonomy for cross-subject EEG emotion recognition, illustrating the five major paradigms.

The contributions of this paper are as follows:

- A novel taxonomic system: We propose a mechanism-driven classification system that synthesizes existing methods into five paradigms: statistical and adversarial distribution alignment, topological and structural modeling, advanced representation learning, generative modeling and style reconstruction, and multimodal complementary fusion. This provides researchers with a clear theoretical map. To present this new taxonomy clearly and structurally, Figure 1 illustrates the systematic framework proposed in this review.
- Critical analysis: Beyond merely listing accuracy improvements, we conduct an in-depth analysis of the risks of negative transfer and the trade-offs between static alignment and dynamic adaptation.
- Future roadmap: We highlight emerging trends such as Source-Free Domain Adaptation (SFDA) and causal inference, providing actionable directions for future research.

The organization of this paper is as follows: Section 2 details the selection strategy and evaluation criteria for the relevant literature; Section 3 introduces mainstream public datasets and evaluation norms for cross-subject tasks; Section 4 provides a detailed analysis of the five categories of cross-subject generalization methods based on the proposed framework; Section 5 systematically compares different categories of methods in terms of data dependency and complexity; Section 6 summarizes the challenges and future trends in the field; finally, Section 7 concludes the paper.

## 2 Methods

To ensure a systematic review of the recent advancements in the field of cross-subject EEG-based emotion recognition, this survey focuses on the retrieval and selection of relevant literature published between January 2020 and December 2025. The literature search covered multiple authoritative academic databases, including Web of Science, IEEE Xplore, and PubMed. The search strategy was constructed based on Boolean logic, with core keywords centered on around “EEG,” “Emotion Recognition,” and “Cross-Subject,” as well as the synonymous expression “Subject-Independent.” Related terms such as “domain adaptation,” “domain generalization,” and “transfer learning” were also considered to improve retrieval coverage.

The initial search yielded a total of 826 literature records, including 439 records from Web of Science, 242 records from IEEE Xplore, and 145 records from PubMed. To construct a high-quality review corpus for systematic analysis, we implemented a multi-level screening process. In the preliminary screening stage, titles, abstracts, and keywords were reviewed. During this stage, 500 records were excluded because they were duplicates or review articles, were not related to EEG-based emotion recognition, not focused on cross-subject or subject-independent settings, related to irrelevant physiological modalities or non-emotion-recognition tasks, or clearly outside the methodological scope of this review. After this preliminary screening, 326 papers were retained for full-text assessment.

Subsequently, a fine-grained selection was conducted through full-text review according to the following three core criteria:

- Relevance: The study must explicitly address cross-subject distribution shift, subject-independent EEG emotion recognition, or closely related generalization problems.
- Innovation: The study must demonstrate clear methodological contributions, rather than being a simple stacking of existing models or a purely application-oriented report.
- Performance validation: The study must provide reproducible quantitative comparison results on public or clearly described EEG emotion recognition datasets, such as SEED, SEED-IV, SEED-V, DEAP, DREAMER, or other commonly used datasets.

**Figure 2:**
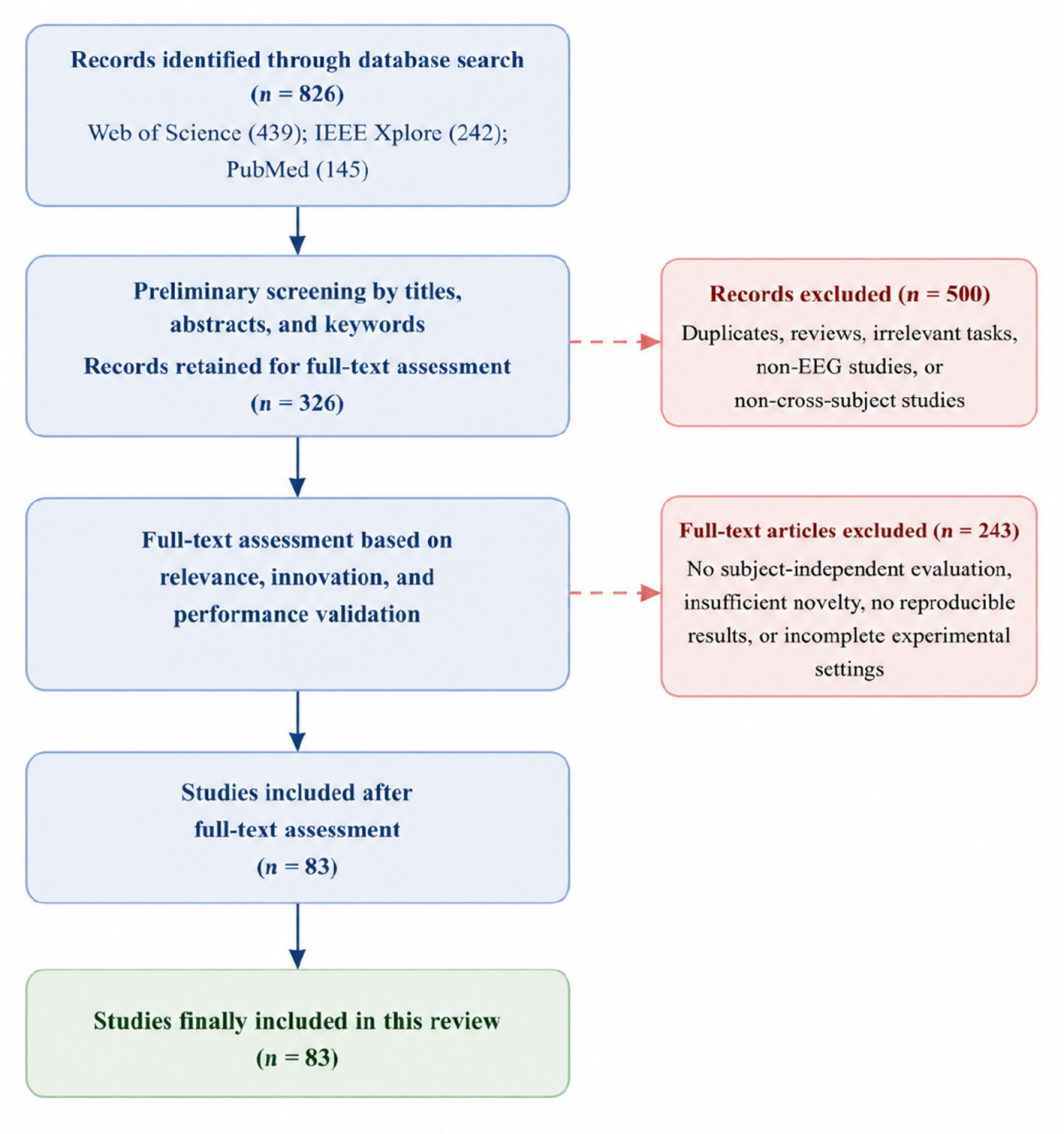
Flowchart of the article screening process.

During the full-text assessment stage, 243 papers were further excluded because they did not provide subject-independent evaluation, lacked sufficient methodological novelty, failed to report reproducible quantitative results, used insufficiently described datasets, or provided incomplete experimental settings. Following this screening process, 83 high-quality papers were ultimately included as the core subjects of analysis for this review. The detailed literature screening process is illustrated in Figure 2.

This review is positioned as a methodological and narrative survey. Therefore, the literature screening process was designed to construct a representative and methodologically relevant review corpus for taxonomy and paradigm-level analysis. To improve the transparency and reliability of the selection process, exclusion reasons were recorded during screening. In addition, the included studies were checked for basic methodological completeness, including whether they clearly reported the dataset, cross-subject evaluation setting, experimental protocol, performance metrics, and reproducible quantitative results.

## 3 Datasets

Research on cross-subject EEG emotion recognition relies heavily on public datasets, whose design methodologies, annotation strategies, and subject scales directly influence the evaluation of model generalization. Currently, mainstream research is primarily conducted on a few classic datasets, most notably DEAP (Koelstra et al., 2012), the SEED series (Zheng and Lu, 2015), and DREAMER (Katsigiannis and Ramzan, 2018). The DREAMER dataset has been increasingly introduced into cross-subject studies in recent years, particularly in multimodal fusion research.

The DEAP dataset collected physiological signals from 32 healthy participants (gender-balanced) stimulated by music videos. Each participant watched 40 1-min video clips. The protocol for each trial consisted of a 3-second baseline recording, followed by the video viewing, after which participants rated their current emotional state using the Self-Assessment Mannequin (SAM) on a 9-point scale across four dimensions: Valence, Arousal, Dominance, and Liking. Throughout the experiment, 32-channel EEG signals were synchronized with various peripheral physiological signals, such as Galvanic Skin Response (GSR) and Electromyography (EMG), constituting a multimodal dataset with continuous emotion dimension labels.

The SEED series datasets (SEED, SEED-IV, and SEED-V) were constructed by the BCMI Laboratory at Shanghai Jiao Tong University. The SEED dataset involves 15 subjects and utilizes Chinese movie clips to induce three categories of discrete emotions: positive, neutral, and negative. SEED-IV extends SEED by increasing the emotion categories to four, namely happiness, sadness, fear, and neutral, while including 15 subjects and using movie clips as stimuli. SEED-V further refines the emotion categories to five (adding disgust) and introduces music videos as new emotion-inducing materials to investigate the effects of multimodal stimuli, with a subject scale of 16. The entire series employs high-density 62-channel EEG equipment for data acquisition.

The DREAMER dataset was collected from 23 subjects. During the experiment, subjects watched 18 audio-visual movie clips designed to induce emotions. Immediately following each stimulus presentation, subjects rated their emotional states on a scale of 1 to 5 across three dimensions: Valence, Arousal, and Dominance. Data acquisition utilized the 14-channel EPOC wireless EEG device to record EEG signals, synchronized with Electrocardiogram (ECG) and GSR peripheral signals. Table 1 compares the similarities and differences among these datasets.

**Table 1:** Similarities and differences of datasets.

Dataset	Subjects	Stimuli	EEG channels	Emotion labels/dimensions
DEAP	32	40 music videos	32	Valence, arousal, dominance, liking
SEED	15	Chinese movie clips	62	Positive, neutral, negative
SEED-IV	15	Chinese movie clips	62	Happiness, sadness, fear, neutral
SEED-V	16	Movie clips	62	Happiness, sadness, fear, disgust, neutral
DREAMER	23	Audio-video clips	14	Valence, arousal, dominance

## 4 Classification and methods

### 4.1 Statistical and adversarial distribution alignment

The core challenge in cross-subject EEG emotion recognition lies in the significant physiological differences and non-stationarity among different subjects, which cause the same emotion category to exhibit distinct distribution patterns in the feature space. To mitigate the resulting domain shift, researchers have widely introduced Domain Adaptation (DA) paradigms (Jayaram et al., 2016; Sun and Saenko, 2016), aiming to enhance cross-subject generalization by aligning the feature distributions of the source and target domains. Existing methods can be broadly categorized into two types: statistical distribution alignment and adversarial distribution alignment. These two approaches differ in their alignment objectives, implementation mechanisms, and robustness. Table 2 summarizes and compares recent key works and model frameworks in cross-subject emotion recognition.

**Table 2:** Summary of literatures related to the alignment of statistics and confrontation distribution.

References	Dataset	Accuracy (%)					
		SEED	SEED-IV	SEED-V	DEAP		DREAMER
					Arousal	Valence	
Chen S. et al. (2025)	SEED+SEED-IV	93.78	78.93	–	–		–
Jimnez-Guarneros and Fuentes-Pineda (2023)	SEED+SEED-IV	93.55	87.96	–	–		–
Zhu et al. (2024)	SEED+SEED-IV+SEED-V	83.21	52.12	60.17	–		–
Li Z. et al. (2022)	SEED+SEED-IV	91.08	81.58	–	–		–
Gu et al. (2024)	SEED+SEED-IV	90.08	77.55	–	–		–
Zhang R. et al. (2024)	SEED+SEED-IV	87.51	68.79	–	–		–
She et al. (2025)	SEED+SEED-IV+SEED-V+DEAP	88.68	67.61	65.57	65.33 (avg)		–
Zhu L. et al. (2026)	SEED+SEED-IV	87.14	63.24	–	–		–
Wang Z. et al. (2024)	DEAP	–	–	–	68.67	67.11	–
Li X. et al. (2024)	SEED+SEED-IV	91.77	75.12	–	–		–
Chen Y. et al. (2025)	SEED+SEED-IV	90.69	74.35	–	–		–
Shirkarami and Mohammadzade (2023)	SEED	84.30	–	–	–		–
Farokhah et al. (2023)	DEAP	–	–	–	94.16	94.81	–
She et al. 2023b)	SEED+SEED-IV+DEAP	86.16	59.29	–	65.59 (avg)		–
Chen S. et al. (2025)	SEED+SEED-IV	93.78	78.93	–	–		–
Dan et al. (2025)	SEED+SEED-IV	93.18	75.36	–	–		–
Tao et al. (2023)	SEED+DEAP	82.05	–	–	77.62 (avg)		–
Xu et al. (2024)	SEED	88.19	–	–	–		–
Lu et al. (2023)	SEED+SEED-IV	93.37	82.32	–	–		–
Huang et al. (2022)	DEAP	–	–	–	63.85 (avg)		–
Xiao et al. (2025)	SEED+SEED-IV+DEAP	89.87	83.90	–	66.13 (avg)		–

The reported accuracies are extracted from the corresponding original studies. Since different studies may adopt different dataset splits, subject-independent validation protocols, cross-session settings, target-domain accessibility assumptions, hyperparameter-tuning procedures, and statistical reporting practices, these results should be interpreted as reported empirical evidence rather than directly comparable values under a unified benchmark. This note applies to Tables 2–6.

#### 4.1.1 Statistical distribution alignment methods

Statistical methods typically achieve cross-domain alignment by minimizing the discrepancy between the source and target domains under specific statistical metrics [e.g., Maximum Mean Discrepancy (MMD), subspace distance, or class-conditional divergence]. The key to these methods lies in selecting the appropriate alignment granularity and objects. Early research found that aligning only marginal distributions often neglects class structure, leading to class confusion. To address this deficiency, numerous studies have introduced the concept of Joint Distribution Adaptation (JDA).

Among them, Chen S. et al. (2025) proposed the MSS-JDA (Multi-Source Self-Selected Joint Domain Adaptation) method. Within a multi-source transfer framework, they introduced a self-selection mechanism that constructs independent branches for each source domain and achieves joint distribution alignment through one-to-one association, thereby suppressing negative transfer caused by poor transferability of certain source domains. Jimnez-Guarneros and Fuentes-Pineda (2023) proposed a Semi-Supervised Domain Adaptation (SSDA) framework. Building on JDA, they incorporated unlabeled samples from the target domain and utilized consistency constraints and class structure preservation to make joint distribution alignment more stable in cross-subject scenarios. Zhu et al. (2024) proposed the JD-IRT (Joint Distributed Instances Represent Transfer) method, which embeds Joint Distribution Deep Adaptation (JDDA) into a deep network structure. This method synchronously aligns marginal and conditional distributions within a multi-layer feature space and combines an Instance-based Resampling Transfer (I-RT) strategy to filter out non-transferable samples, effectively mitigating interference caused by inter-subject variability.

Since target domain labels are unknown, class-conditional alignment relies heavily on the quality of pseudo-labels; direct fine-grained alignment can easily introduce noise. Consequently, some studies have proposed dynamic alignment strategies. Li Z. et al. (2022) proposed the DDA (Dynamic Domain Adaptation) method, which decomposes cross-domain differences into global domain shifts and intra-class sub-domain shifts. It adopts a step-by-step alignment strategy from global to local during training, thereby reducing the risks associated with unreliable early-stage pseudo-labels. Gu et al. (2024) proposed the CL-DDA (Collaborative Learning and Dynamic Distribution Adaptation) method, which builds a collaborative learning sub-network to dynamically align global marginal distributions and local sub-domain distributions simultaneously, addressing the issue of insufficient generalization in multi-source transfer scenarios. Zhang R. et al. (2024) designed the MGFKD (Multi-domain Geodesic Flow Kernel Dynamic Distribution Alignment) method from a geometric perspective. This approach introduces dynamic alignment into the Grassmann manifold space, combining the Geodesic Flow Kernel (GFK) with knowledge transfer mechanisms to significantly improve the stability of cross-subject emotion recognition.

To avoid incorrectly pulling samples of different classes closer during alignment, increasing attention has been paid to sub-domain or class-conditional distribution alignment. She et al. (2025) proposed the DFSAN (Dual Filtration Sub-domain Adaptation Network) method, which evaluates transferability between source and target subjects to select high-quality source domains and performs metric alignment at the sub-domain level, effectively suppressing negative transfer. Zhu L. et al. (2026) proposed the MS-SAMCC (Multi-Source Sub-domain Adaptation and Minimum Class Confusion) method, decomposing multi-source alignment into global distribution alignment and intra-sub-domain alignment, while introducing a Minimum Class Confusion (MCC) regularization term to maintain inter-class separability. Wang Z. et al. 2024) proposed the CARL-DSAN method, which models the structure of the emotion space by incorporating cerebral asymmetry features and implements class-conditional sub-domain alignment via a Deep Subdomain Adaptation Network (DSAN), achieving more discriminative representations in cross-subject tasks.

Considering the significant spatial structure and channel dependency of EEG signals, some studies combine distribution alignment with structural modeling. The Gusa (Graph-based Unsupervised Sub-domain Adaptation) method, proposed by Li X. et al. (2024), models cross-subject alignment as an unsupervised sub-domain adaptation problem on graph structures, achieving fine-grained alignment through multi-level constraints (node-wise, channel-level, and emotion-level). Chen Y. et al. (2025) proposed a Multisource Structural Deep Clustering method, which introduces structure preservation and distribution regularization in the latent space to guide target domain samples into clear sub-domain clusters, thereby mitigating heterogeneity across subjects and sessions.

Another category of methods posits that cross-subject differences are difficult to align directly in the original feature space; therefore, they first construct a subspace more suitable for transfer. Shirkarami and Mohammadzade (2023) proposed an Enhanced Subspace Alignment method with clustering and weighting, which clusters source subjects to select appropriate source data, focusing the alignment process on source sub-groups most similar to the target. Farokhah et al. (2023) designed a robust Unified Domain Adaptation framework (specifically MFA-LR), pointing out that different subjects do not fully share a feature space. They proposed modeling both shareable and non-shareable components at the subject and class levels to avoid over-alignment.

In multi-source emotion recognition scenarios, differences between source domains further amplify transfer difficulty. She et al. (2023b) proposed a Multi-source Associate Domain Adaptation method, realizing joint modeling of cross-subject and cross-session data by simultaneously learning domain-invariant and domain-specific features. Chen S. et al. (2025) (in their MSS-JDA work) utilized a multi-source branch mechanism to control alignment objects, embodying the philosophy of “selective alignment” in multi-source scenarios. Furthermore, to enhance the robustness of statistical alignment against noise and mean drift, Dan et al. (2025) proposed the DADPc (Domain Adaptive Deep Possibilistic Clustering) method. This approach combines distribution alignment with possibilistic clustering, introducing fuzzy entropy regularization and a weighted loss function to achieve stable cross-domain alignment even under uncertain pseudo-labels.

Beyond traditional DA, some research revisits distribution alignment from the perspective of Domain Generalization (DG). Tao et al. (2023) proposed the LDG (Local Domain Generalization) method, which divides samples into multiple local sub-domains. Through low-rank constraint reconstruction and local correlation modeling, it prioritizes aligning local structures most relevant to the target domain. Some works do not alter the alignment objective itself but indirectly reduce distribution discrepancies through more robust feature representations. Xu et al. (2024) proposed MASTF-net, combining spatiotemporal image-like features with frequency domain features to provide a more stable input representation for cross-subject alignment. Lu et al. (2023) proposed a Hybrid Transfer Learning strategy (specifically DFF-Net), emphasizing a strategy of “learning transferable representations first, then performing individual adaptation” by conducting DA pretraining before transferring to the target subject.

#### 4.1.2 Adversarial distribution alignment methods

Unlike statistical methods, adversarial approaches introduce domain discriminators or generative models to learn domain-invariant or domain-variant representations through a game-theoretic process. Huang et al. (2022) proposed the GDAKF (Generator-based Domain Adaptation Method with Knowledge Free) method, employing Generative Adversarial Networks (GANs) to map source domain features into the target domain distribution space. It maintains emotion-related information through additional constraints, offering the advantage of explicitly learning the cross-subject distribution transformation function. Xiao et al. (2025) proposed the ACAN (Auxiliary Classifier Adversarial Networks) method. This approach introduces a task classifier and a maximum subdomain discrepancy constraint during adversarial training, ensuring the adversarial alignment process is guided by class-discriminative information, thereby mitigating misalignment issues in cross-subject emotion recognition.

### 4.2 Topological and structural modeling

In addition to distribution alignment strategies, another significant research avenue in cross-subject EEG emotion recognition involves explicitly modeling the spatial topological structure and spatiotemporal dependencies of EEG signals. Unlike methods that treat EEG channels as independent features, topological and structural modeling emphasizes functional connectivity, information flow between brain regions, and their inter-subject variability. This approach is crucial for enhancing cross-subject generalization capabilities.

Addressing the issue that fixed brain connectivity structures struggle to adapt to individual differences, numerous studies have proposed constructing graph structures dynamically in a data-driven manner. Chen R. et al. (2025) proposed a Progressive Multi-Domain Adaptive Network, which utilizes a reinforcement learning-driven self-constructing graph module. This allows the model to automatically learn optimal connection relationships among EEG channels in cross-subject scenarios, enabling the graph structure to evolve progressively during the domain transfer process, thereby maintaining the stability of emotion-related topological information while aligning distributions. Similarly, Pan et al. (2024) proposed the ST-SCGNN method, which extends the self-constructing graph concept to spatiotemporal dimensions. By dynamically updating the graph structure based on input signals, they validated the effectiveness of dynamic graph structures in extreme low-sample cross-domain scenarios for both emotion recognition and Disorders of Consciousness (DOC) detection. These studies collectively demonstrate that self-constructing graph mechanisms can mitigate the mismatch problem of manual prior topologies in cross-subject settings, representing a key trend in recent structural modeling.

Beyond directly constructing graph structures, some research attempts to jointly design dynamic graph convolution (DGC) and domain adaptation strategies. An et al. (2024) proposed the IDDA method, which utilizes DGC to dynamically learn intrinsic relationships between EEG channels and optimizes it jointly with Dynamic Domain Adaptation (DDA). This achieves structural alignment at the emotion sub-domain level while aligning global domain distributions, thereby enhancing the discriminability of cross-subject recognition. Wang and Zhao (2025) adopted a similar “coarse-to-fine” philosophy. On the basis of dynamic graph structures, they introduced dynamic factors to transition the model training process from global structural consistency to local sub-domain structural alignment, effectively alleviating structural drift under cross-session and cross-subject conditions. A common characteristic of these methods is that they treat structural modeling as an integral part of the domain transfer process, rather than an independent module.

Addressing the limitations of traditional Graph Convolutional Networks (GCNs) that rely on static, symmetric, and fully connected graph structures, researchers have begun to introduce structural constraints that align better with neurophysiological mechanisms. Shen et al. (2025) proposed DSDirGCN-AM, which explicitly models directional information flow between brain regions via Dynamic Sparse Directed Graph Convolution and combines a channel attention mechanism to suppress redundant connections, making the constructed graph structure closer to real neural transmission patterns. Zhong et al. (2022) proposed RGNN (Regularized GNN), which starts from brain organizational structure. It utilizes a biologically inspired adjacency matrix to constrain graph connection sparsity and enhances cross-subject robustness through Node-level Domain Adversarial Training (NodeDAT), embodying a modeling approach that combines neural priors with structural learning. These methods emphasize that structural rationality itself is a vital factor in improving cross-subject generalization.

To enhance the modeling capability of key brain regions and their interactions, several studies have introduced Graph Attention Mechanisms (GAT). Li Z. et al. (2023) proposed STFCGAT, which fuses single-channel Differential Entropy (DE) features with cross-channel functional connectivity features and highlights emotion-relevant connections through a multi-head graph attention mechanism, achieving significant performance improvements in cross-subject experiments. Li J. et al. (2023) proposed STGATE, which further combines a Transformer encoder to extract time-frequency features, followed by a dynamic graph attention network to learn inter-channel dependencies, validating the effectiveness of the Transformer and GNN architecture in cross-subject emotion recognition. Both works suggest that attention mechanisms help mitigate interference from irrelevant brain regions in cross-subject scenarios.

Some studies argue that a single structure is difficult to fully characterize the complexity of EEG signals, thus adopting multi-branch or hybrid structures. Xu et al. (2025) proposed DDNet, which models local temporal patterns and global topological relationships in parallel through a multi-head attention branch and a dynamic graph convolution branch, significantly improving the model's adaptability to individual differences. Li W. et al. (2024) proposed DSSN (Dynamic Structure Selection Network). From the perspective of structural selection, it constructs three feature streams—spatial, temporal, and spatiotemporal, and matches the most suitable structural branch for different subjects through a dynamic selection mechanism, thereby avoiding performance degradation caused by a unified structure in cross-subject scenarios.

Addressing the issue that different subjects may correspond to different functional brain region patterns, researchers have begun to introduce specialized structural modeling (or Mixture of Experts). Liu X.-H. et al. (2024) proposed the MoGE (Mixture of Graph Experts) model, which partitions EEG channels and assigns them to different graph expert networks. This allows each expert to focus on modeling specific functional areas, achieving excellent cross-subject generalization without explicit domain alignment. This reflects the philosophy of structural decoupling as an alternative to distribution alignment. In multi-modal emotion recognition scenarios, structural modeling must also address modality heterogeneity. Huang et al. (2024) proposed CMMGD (Correlation-Metric-based Multi-modal Graph Decomposition). Through a correlation-driven graph decomposition strategy, it splits the multimodal hybrid graph into consistent subgraphs and discrepant subgraphs, enabling the model to focus on structural subspaces that are stably activated across modalities and subjects. This provides a new structural modeling paradigm for multimodal cross-subject emotion recognition. Additionally, Liu K. et al. (2025) proposed the ASJDA (Adaptive Source Joint Domain Adaptation) method, which, after selecting relevant source domains, performs joint alignment at the domain and class-sub-domain levels supported by graph neural networks.

Some works, while centered on domain adaptation, rely on structure-aware feature extraction for performance gains. Lu et al. (2024) proposed DSP-EmotionNet, which explicitly introduces channel topological relationships within a CNN framework through a Spatial Activity-Topology Joint Feature Extraction Module (SATFEM), providing more stable structured feature representations for subsequent cross-subject domain adaptation. Similarly, MS-FRAN (Li et al., 2023b) and MS-AGDA (Liu J. et al., 2025) (proposed by Liu et al.), while focusing primarily on multi-source distribution alignment, utilize wide feature extractors and gating mechanisms that essentially perform implicit modeling of structural features for different subjects, thereby mitigating the impact of structural mismatch on transfer efficacy.

In conclusion, topological and structural modeling methods characterize the spatial and spatiotemporal dependencies of EEG signals from different levels through strategies such as self-constructing graphs, dynamic graph convolution, attention mechanisms, multi-branch structures, and specialized modeling. Recent research trends are gradually shifting from static prior topologies to data-driven, evolvable, and structurally selectable graph modeling approaches. These are deeply integrated with domain adaptation and multimodal learning, providing a more robust modeling foundation for cross-subject EEG emotion recognition. Table 3 summarizes key works and model frameworks in this direction.

**Table 3:** Literature review of topology and structure modeling.

References	Dataset	Accuracy (%)					
		SEED	SEED-IV	SEED-V	DEAP		DREAMER
					Arousal	Valence	
Chen R. et al. (2025)	SEED+SEED-IV	97.03	88.18	–	–		–
Pan et al. (2024)	SEED+SEED-IV	85.90	76.37	–	–		–
An et al. (2024)	SEED+SEED-IV	85.75	72.36	–	–		–
Wang and Zhao (2025)	SEED+SEED-IV	89.76	65.28	–	–		–
Shen et al. (2025)	SEED+SEED-IV	93.19	81.30	–	–		–
Zhong et al. (2022)	SEED+SEED-IV	85.30	73.84	–	–		–
Li Z. et al. (2023)	SEED+DEAP	94.83	–	–	91.19	92.03	–
Li J. et al. (2023)	SEED+SEED-IV+DREAMER	90.37	76.43	–	–		76.35
Xu et al. (2025)	SEED+SEED-IV	92.63	85.03	–	–		–
Liu X.-H. et al. (2024)	SEED+SEED-IV+SEED-V	88.00	74.30	81.80	–		–
Liu K. et al. (2025)	SEED+SEED-IV+DEAP	96.81	89.69	–	69.31 (avg)		–
Lu et al. (2024)	SEED+SEED-IV	82.50	65.90	–	–		–
Liu J. et al. (2025)	SEED+SEED-IV+DEAP	92.54	85.86	–	65.59 (avg)		–

### 4.3 Advanced representation learning

Beyond directly aligning the distributions of source and target domains, constructing advanced feature representations that are highly discriminative, noise-resistant, and aligned with neurophysiological mechanisms constitutes another critical pathway for enhancing the robustness of cross-subject EEG emotion recognition. In recent years, researchers have proposed various strategies to enhance feature transferability by combining contrastive learning, semi-supervised and source-free transfer, generative reconstruction, and novel deep architectures. Table 4 summarizes the relevant literature on advanced representation learning methods.

**Table 4:** A literature review of advanced representation learning methods.

References	Dataset	Accuracy (%)					
		SEED	SEED-IV	SEED-V	DEAP		DREAMER
					Arousal	Valence	
Zhu Q. et al. (2026)	SEED	89.61	–	–	–		–
Zhang Y. et al. (2024)	SEED+SEED-IV+DEAP	>99	>99	–	97.13 (avg)		–
Alghamdi et al. (2025)	SEED	97.70	–	–	–		–
Chang et al. (2025)	SEED	89.30	–	–	–		–
Liu H. et al. (2024)	DEAP	–	–	–	58.10	61.10	–
Ouyang et al. (2025)	SEED+DEAP	84.39	–	–	79.75	89.01	–
Ran et al. (2024)	SEED+SEED-IV	86.44	82.81	–	–		–
Zhou R. et al. (2025)	SEED+SEED-IV+SEED-V	91.35	65.53	62.75	–		–
Zhang et al. (2025)	SEED	95.86	–	–	–		–
Zhong et al. (2025)	SEED+SEED-IV+SEED-V	96.41	82.20	84.70	–		–
Zhou B. et al. (2025)	SEED+SEED-IV+DEAP	85.90	70.40	–	65.30 (avg)		–
Li J. et al. (2022)	SEED+DEAP	87.05	–	–	71.92	71.29	–
Gong et al. (2024)	SEED	96.92	–	–	–		–
Ding et al. (2025)	SEED	80.20	–	–	–		–
Ye et al. (2022)	DEAP	–	–	–	98.85	98.72	–
Li et al. 2023a)	SEED+DEAP	83.43	–	–	63.68	60.51	–

#### 4.3.1 Contrastive learning and self-supervised representations

Contrastive Learning (CL), which works by pulling positive sample pairs closer while pushing negative pairs apart, has become a mainstream paradigm for mining cross-subject invariant features. To address the interference caused by individual differences, Zhu Q. et al. (2026) proposed the CLAE model. This model integrates an attention mechanism into a convolutional network and extracts subject-invariant features by maximizing the similarity of EEG representations from different subjects under the same stimulus. Zhang Y. et al. (2024) combined contrastive learning with Graph Convolutional Networks (GCN) to construct the CLGCN model, which generates a standardized brain network learning matrix to reveal commonalities in brain connectivity while decoding emotional states. Addressing the difficulty of traditional CL in handling complex hierarchical relationships, Shao et al. (2025)—although primarily focusing on identity recognition—proposed an Interpretable Contrastive Learning Transformer. Their approach of capturing state-invariant features by constructing a cross-paradigm alignment loss provides a valuable reference for emotion feature disentanglement.

Considering the complex hierarchical structure of EEG data, some studies have shifted to non-Euclidean spaces. Alghamdi et al. (2025) introduced Cross-Subject Contrastive Learning (CSCL) in a hyperbolic space, leveraging the advantages of hyperbolic geometry to effectively capture the hierarchical patterns of brain regional signals. Chang et al. (2025) further proposed Multi-Scale Hyperbolic Contrastive Learning (MSHCL), which applies contrastive losses at both emotion and stimulus scales to learn more robust subject-invariant representations.

Recently, Wan et al. (2025) further connected data generation with invariant representation learning for EEG-based emotion recognition. They proposed a generative pre-trained Transformer framework, EEGPT, to generate time-invariant components from raw EEG signals, and introduced a Contrastive Learning method for Time-invariant and Subject-invariant EEG data generation (CLTISI). Unlike conventional augmentation strategies that mainly expand the sample size, this method explicitly aims to extract time-invariant and subject-invariant components, thereby improving robustness against EEG non-stationarity and inter-subject variability. This work suggests that generative modeling and contrastive learning are not isolated paradigms; rather, they can be integrated to construct more transferable EEG representations for cross-subject emotion recognition.

Inspired by neural representation mechanisms in the visual cortex, Li D. et al. (2025) designed the Parallel Contrastive Multi-Source Domain Adaptation (PCMDA) model. This model mimics the brain's self-supervised learning mechanisms, utilizing two-stage alignment and self-attention to extract frequency band weights. Liu H. et al. (2024) exploited the non-stationarity of EEG signals to construct a self-supervised pretext task, proposing a negative segment selection algorithm to reduce noise interference in unlabeled data. Additionally, Ouyang et al. (2025) proposed a Dual-Branch Self-Supervised Framework that achieves mutual learning between time-domain and time-frequency domain representations, significantly reducing dependency on labeled data. In terms of integration with domain adaptation, Ran et al. (2024) proposed a “coarse-to-fine” strategy, introducing local contrastive learning on top of global MMD alignment to separate heterogeneous samples within different sub-domains.

#### 4.3.2 Semi-supervised, source-free, and meta-learning strategies

Addressing the issues of label scarcity in the target domain and privacy protection, researchers have proposed various advanced strategies utilizing unlabeled data and source models.

To fully leverage unlabeled data, Zhou R. et al. (2025) proposed the EEGMatch framework. By combining EEG-Mixup data augmentation with two-step pairwise learning (prototype-level and instance-level), it alleviates the label scarcity problem. Zhang et al. (2025) designed the SASD-MCL model, which effectively fuses labeled and unlabeled data through hybrid contrastive data augmentation and semi-supervised alignment self-distillation. Zhong et al. (2025) focused on the quality of pseudo-labels, proposing the DAPLP (Domain Adaptation via Pseudo-Label Propagation) method. It generates pseudo-labels through global alignment and optimizes the label propagation process using smoothing techniques, achieving unsupervised cross-domain adaptation.

Addressing privacy scenarios where source data is invisible, Zhou B. et al. (2025) pioneered the ProDG strategy. This approach utilizes high-confidence predictions from the source model to construct a “proxy domain,” achieving Source-Free Domain Adaptation (SFDA) by maximizing mutual information. In the realm of meta-learning, Li J. et al. (2022) combined multi-scale residual networks with Meta-Transfer Learning (MTL), utilizing the rapid adaptation capability of meta-learning to significantly narrow individual differences. Gong et al. (2024) addressed multimodal (EEG and eye movements) scenarios by proposing Completeness-induced Adaptive Broad Learning (CiABL), which avoids spurious correlations between modalities through a weighted alignment mechanism.

#### 4.3.3 Spatiotemporal joint and hybrid deep architectures

To capture the spatiotemporal dynamics of EEG more precisely, various novel network architectures have been applied to cross-subject tasks. Ding et al. (2025) proposed EmT (Emotion Transformer), which converts EEG into graph sequences via a temporal graph construction module and utilizes a temporal context Transformer to capture long-term dependencies. Ye et al. (2022) designed MLBNet, which contains two learners with temporal bias and spatial bias, respectively. Through Deep Spatiotemporal Mutual Learning, the two learners imitate each other's prediction probabilities, improving performance without requiring a large teacher model. Li et al. (2023a) proposed the STCL model, which utilizes MLP and Transformer encoders for spatial and temporal constraint learning, respectively, and further reduces domain discrepancies through adversarial training.

### 4.4 Generative modeling and style reconstruction

Compared to discriminative methods that merely reduce inter-domain distances in the feature space, generative modeling offers a more interpretable and reconstructible solution for cross-subject emotion recognition by explicitly learning the latent distributions of EEG data. Relevant works can be broadly categorized into three paradigms: latent variable reconstruction and generative augmentation, graph structure disentanglement and style transfer, and distillation-based domain generalization and cross-domain knowledge transfer. Table 5 summarizes and compares cross-subject emotion recognition studies in this direction over recent years.

**Table 5:** Literature review of generative modeling and style reconstruction methods.

References	Dataset	Accuracy (%)					
		SEED	SEED-IV	SEED-V	DEAP		DREAMER
					Arousal	Valence	
Li X. et al. (2020)	SEED+DEAP	85.81	–	–	79.89	76.23	–
Wang Y. et al. (2024)	SEED+SEED-IV	88.27	72.70	–	–		–
Quan et al. (2023)	SEED+SEED-IV	92.83	79.30	–	–		–
Li R. et al. (2023)	DEAP+DREAMER	–	–	–	73.28	71.33	83.33 (Arousal) 80.43 (Valence)
Ahuja and Sethia (2025)	SEED	87.75	–	–	–		–
Chen et al. (2024)	SEED+SEED-IV	92.54	75.65	–	–		–

#### 4.4.1 Latent variable decoding and reconstruction

Addressing the issues of high noise, non-stationarity, and significant inter-subject variability in EEG, numerous studies utilize generative models such as Variational Autoencoders (VAEs) or Autoencoders to compress emotion-discriminative information into a stable latent space, while suppressing subject-specific noise via reconstruction losses. On one hand, VAEs serve as unsupervised latent variable modelers to extract more “emotion-relevant” latent variables from multi-channel EEG. For instance, Li X. et al. (2020) were the first to introduce VAEs into multi-channel EEG decoding, learning latent emotion factors through an unsupervised generative model to achieve natural filtering of individual differences. On the other hand, Wang Y. et al. (2024) proposed DMMR, employing a multi-decoder Denoising Mixing Mutual Reconstruction strategy. By enforcing mutual reconstruction, the encoder is compelled to capture “common features among similar subjects,” thereby enhancing cross-subject stability. Quan et al. (2023) proposed MR-VAE, which combines generative latent representation learning with multi-source selection and transfer strategies, enabling the model to automatically mine emotion-related features from multi-channel EEG and reduce negative transfer. Li R. et al. (2023) proposed MTLFuseNet, which fuses unsupervised spatiotemporal latent features learned by a VAE with supervised spatiotemporal-frequency features learned by a GCN-GRU. It reinforces inter-class separation through focal loss and triplet center loss under a multi-task learning framework, making the fused representations more discriminative in cross-subject recognition. Additionally, Bethge et al. (2022) proposed an adversarial inference method that constrains the latent space from another perspective: explicitly suppressing data-source-correlated information leakage during multi-source learning to obtain purer domain-invariant representations.

In summary, the commonality of these methods lies in using the strong constraint of “generation-reconstruction” to squeeze emotion information into the latent space while pushing subject noise back into the reconstruction error, mechanistically enhancing cross-subject transferability.

#### 4.4.2 Generative augmentation

Beyond improving transferability via “latent space constraints,” another route is to directly complete or augment target domain style data using generative models to mitigate the dual problems of training sample scarcity and excessive cross-domain discrepancies. In the Generative Adversarial Network (GAN) lineage, Chang et al. (2024) proposed CS-GAN, combining Compressed Sensing (CS) with GANs. It learns the mapping from speech to “listener EEG” to generate virtual EEG and ERP signals usable for emotion discrimination, thereby indirectly improving cross-subject robustness. In contrast, the more recent Diffusion Model lineage emphasizes “more stable and controllable” generation. Ahuja and Sethia (2025) proposed TRANSIT-EEG, utilizing Personalized Diffusion Probabilistic Models (IDPM) to generate subject-specific synthetic data to resist artifact interference. It further combines a Self-Organized Graph Attention Transformer (SOGAT) with Low-Rank Adaptation (LoRA) to achieve rapid adaptation to new subjects.

#### 4.4.3 Graph disentanglement and style transfer

When EEG is explicitly modeled as a graph structure (channels/regions as nodes, connections as edges), cross-subject differences often manifest as dual shifts in graph structure and graph representation. Consequently, researchers have begun to introduce the concept of “Content-Style Separation” into EEG graph modeling, achieving cross-domain transfer through disentanglement and recombination.

Closely related studies on brain graph generation also provide useful methodological insights for cross-subject EEG emotion recognition. For example, Zuo et al. (2023) proposed a distribution-regularized adversarial graph autoencoder with Transformer (DAGAE) for dementia diagnosis. This model generates brain functional networks by regularizing the latent distribution and using a Transformer generator to capture long-range dependencies among highly correlated brain regions. Although this study focuses on dementia diagnosis rather than emotion recognition, its core idea is highly relevant to EEG-based affective computing: graph generation can be used not only for data augmentation but also for preserving topological properties and discriminative connectivity patterns in brain networks.

One representative category is Graph Domain Disentanglement. Chen et al. (2024) proposed GDDN, which explicitly separates commonality and specificity components within EEG graph connections and representations, enabling the model to stably preserve emotion-related structures while suppressing individual differences. Another category is Cross-Dataset Style Transfer. Zhou Y. et al. (2025) proposed E^2^STN, which recombines the “emotion content” of the source domain with the “style features” of the target domain, achieving distribution alignment and transferable representation learning across datasets. Addressing the common issue of label space/class inconsistency in cross-dataset scenarios, Jiang et al. (2023) further proposed a Domain Symmetric Module and a Prediction Balancing Module to reduce uncertainty propagation and stabilize the transfer process. Similarly, Li J. et al. (2020) offered a more direct “style mapping” perspective. Based on multi-source transfer learning, they perform source-domain selection via a small amount of calibration data and employ style transfer mapping to explicitly reduce the distribution difference between target and source domains, supporting rapid deployment for new users.

Overall, the core contribution of graph disentanglement and style transfer lies in conceptualizing cross-subject variability not as noise that must be eliminated entirely, but as “style.” By structurally separating it from “emotion content” and then performing recombination alignment, the transfer process becomes more controllable.

#### 4.4.4 Distillation-based domain generalization

When the target domain is inaccessible (due to privacy, cross-dataset constraints, or real-world deployment), another important branch of generative modeling is Knowledge Distillation and Domain Generalization (DG). These methods transfer discriminative emotion knowledge learned by the source domain to a lighter, more generalized model via Teacher-Student networks, often using self-distillation to maintain representation stability.

Li W. et al. (2025) proposed DBDG as a typical paradigm. This method does not rely on target domain data for training; instead, it utilizes online distillation and self-distillation modules to efficiently transfer the discriminative emotion features from the teacher model to the student model, thereby achieving robust generalization performance on unseen data domains. Such methods complement the aforementioned style transfer approaches: while style transfer tends to “pull the target towards the source,” distillation-based generalization tends to “make the model itself insensitive to domain changes.” Together, they propel cross-subject EEG emotion recognition toward real-world applications.

### 4.5 Multimodal complementary fusion

Multimodal emotion recognition, by fusing internal physiological signals (e.g., EEG) with external behavioral signals (e.g., eye movements), significantly outperforms unimodal methods in terms of information complementarity, noise suppression, and cross-subject generalization. Existing research generally concurs that the key to multimodal complementary fusion lies not in simple feature concatenation, but in the alignment of cross-modal discrepancies and the adaptive selection of complementary information, which is particularly crucial in cross-subject scenarios.

In cross-subject multimodal fusion, some studies focus on the joint alignment of cross-modal and cross-domain distribution shifts. Jimnez-Guarneros and Fuentes-Pineda (2025) proposed MACDB, which effectively enhances robustness on new subjects through intra-modal normalization, inter-modal distance constraints, and joint distribution alignment, combined with EEG perturbation consistency constraints. Furthermore, Jimnez-Guarneros and Fuentes-Pineda ((2024) introduced a “coarse-to-fine” cross-modal alignment strategy in CFDA-CSF. Under unsupervised conditions, this method simultaneously aligns global distributions and modality-specific features, rendering the fused feature space both shared and discriminative.

To avoid negative transfer in multimodal and multi-subject transfer, Jimnez-Guarneros et al. (2025a) approached the problem from a multi-source domain perspective, proposing MMDA. By jointly aligning marginal and conditional distributions and incorporating a cross-modal feature selection mechanism, the model focuses on transferring modality information relevant to the target subject. Similarly, Cheng et al. (2025) explicitly decomposed EEG features into background components and task-related components in CPDAN. By performing adversarial alignment at both global and local levels, they reduced intra-class confusion, providing purer discriminative features for multimodal fusion.

Regarding fusion strategy design, researchers emphasize dynamic modeling of modality complementarity. Chen C. et al. (2025) proposed CMSLNet, utilizing an instance-level robust consistency metric to characterize emotional consistency between EEG and eye movements. Through an attention-based low-rank fusion mechanism, it adaptively integrates multimodal features, selectively enhancing key complementary information. Fu et al. (2023) introduced a multi-scale fusion module in MFFNN, employing cross-channel attention to screen effective modal information across different spatial scales, thereby avoiding information loss associated with single-scale fusion.

Beyond fusion operators, cross-modal interaction and contrastive learning are employed to reinforce complementary relationships. Zhu et al. (2025), within a dynamic adversarial domain adaptation framework, combined self-attention and cross-attention mechanisms to model intra-modal and inter-modal affective features. They introduced a cross-modal contrastive loss to bridge modality heterogeneity, ensuring greater semantic consistency in fused representations. Jimnez-Guarneros et al. (2025b) proposed CMJDA for semi-supervised scenarios. Through cross-modal correlated feature learning and joint distribution alignment, it effectively leverages a small number of labeled target samples to guide the transfer of multimodal complementary information to new subjects.

In terms of deployability, Zhang et al. (2021) proposed CSCM, considering both cross-subject variations and cross-modal differences. By utilizing complementary information from EEG and eye movements during training while relying solely on easily acquirable eye tracking signals during testing, they achieved a transfer from multimodal complementarity to unimodal inference.

Datasets and experimental analyses also validate the effectiveness of multimodal complementarity. Jiang et al. (2025) constructed the SEED-VII dataset (containing EEG and eye movements) and proposed the MAET model, demonstrating the advantages of multimodal fusion in cross-subject generalization under continuous emotion intensity annotation conditions. Furthermore, Liu et al. (2022) discovered through cross-cultural experimental analysis that EEG and eye movements exhibit stable complementary characteristics across different cultural backgrounds. They also revealed asymmetry in cross-cultural transfer, providing empirical evidence for the generalization research of multimodal fusion methods.

It is worth noting that the efficacy of multimodal fusion depends to some extent on the expressive capability of unimodal features. Therefore, several unimodal cross-subject EEG methods provide a solid foundation for multimodal fusion. For instance, Han et al. (2024) enhanced EEG representations through multi-scale temporal modeling and brain topological structures; She et al. (2023a) reduced negative transfer effects from a multi-source domain transfer perspective; Cimtay and Ekmekcioglu (2020); and Lu and Chen (2025) improved cross-subject EEG stability through deep feature learning and signal-level denoising, respectively. The structural-nonstructural dual-stream attention fusion framework by Ye et al. (2025) offers valuable insights for “fusion-as-selection” in multimodal scenarios. Table 6 summarizes and compares cross-subject emotion recognition studies in this direction over recent years.

**Table 6:** References on multimodal complementary fusion methods.

References	Dataset	Accuracy (%)					
		SEED	SEED-IV	SEED-V	DEAP		DREAMER
					Arousal	Valence	
Jimnez-Guarneros and Fuentes-Pineda (2025)	SEED+SEED-IV+SEED-V	86.68	85.03	86.48	–		–
Jimnez-Guarneros and Fuentes-Pineda (2024)	SEED+SEED-IV+SEED-V	93.05	85.72	88.49	–		–
Chen C. et al. (2025)	SEED-IV+SEED-V	–	83.15	87.32	–		–
Fu et al. (2023)	SEED-IV	–	87.32	–	–		–
Zhu et al. (2025)	SEED+SEED-IV+SEED-V	94.96	89.82	89.22	–		–
Jimnez-Guarneros et al. (2025b)	SEED-IV+SEED-V	–	92.50–96.13	92.50–96.13	–		–
Zhang et al. (2021)	SEED+SEED-IV	76.18	72.22	–	–		–
Han et al. (2024)	DEAP	–	–	–	91.31	90.45	–
She et al. (2023a)	SEED+DEAP	86.59	–	–	64.40 (avg)		–
Cimtay and Ekmekcioglu (2020)	SEED+DEAP	86.56	–	–	72.81 (avg)		–
Lu and Chen (2025)	SEED	>98	–	–	–		–

## 5 Method comparison

Based on the taxonomy proposed above, this chapter conducts a systematic horizontal comparison of the five paradigms of cross-subject EEG emotion recognition across four key dimensions: performance, data dependency, physiological interpretability, and computational complexity. Table 7 summarizes the pros and cons of each paradigm.

**Table 7:** Multidimensional comparison of main paradigms of cross-individual EEG emotion recognition.

Classification	Advantage	Limitations	Computational complexity	Representative models
Alignment of statistics and confrontation distribution	Efficient, simple and fast	Semantic misalignment, limited generalization	Low	MSS-JDA
Topology and structure modeling	Strong robustness	Sensitive to graph quality, high overhead	Medium	STFCGAT
Advanced representation learning	Label-efficient, noise-robust	High training cost, slow convergence	Medium/high	CLGCN
Generative modeling and style reconstruction	Alleviates data scarcity	Unstable training, resource-intensive	Extremely high	MTLFuseNet
Multimodal complementary fusion	High accuracy, multimodal fusion	System complexity, synchronization issues	High	MSTimesNet

Prior to comparing these paradigms along specific evaluation dimensions, it is necessary to clarify their conceptual boundaries and interrelationships. These five paradigms do not simply represent different algorithmic families; rather, they address the problem of cross-subject generalization at distinct levels. Statistical and adversarial distribution alignment operates primarily at the feature-distribution level, aiming to reduce domain discrepancy between source and target subjects. Topological and structural modeling shifts the emphasis from distributional similarity to the preservation of brain-region relationships, examining whether emotion-related neural structures remain stable across individuals. Advanced representation learning pushes this abstraction further by seeking subject-invariant yet task-discriminative representations through self-supervised, contrastive, or meta-learning objectives. Generative modeling and style reconstruction treats inter-subject variability as a reconstructible or separable “style” factor, seeking to disentangle emotion-related content from subject-specific patterns. Multimodal complementary fusion extends the problem beyond EEG itself, introducing auxiliary modalities to compensate for the inherent uncertainty and instability of single-modal signals.

Accordingly, these paradigms should be understood as partially overlapping and mutually complementary, rather than mutually exclusive. Distribution alignment provides a basic mechanism for narrowing cross-subject gaps but often requires structural constraints or discriminative representation learning to prevent semantic distortion. Structural modeling enhances physiological plausibility, yet can still benefit from representation learning or generative disentanglement when subject-specific graph patterns exhibit substantial variation. Generative methods can augment or reconstruct transferable features, while multimodal fusion supplies supplementary information during training or inference. From this perspective, the methodological evolution of cross-subject EEG emotion recognition can be characterized as a progressive shift from “aligning distributions” to “preserving semantic structure,” and further toward “disentangling subject-specific factors” and “integrating complementary information.” This conceptual framework lays the foundation for the subsequent comparison across performance, data dependency, interpretability, and computational complexity.

### 5.1 Performance and generalization boundaries

From the perspective of evolutionary trends, the accuracy of cross-subject emotion recognition has shown a stepwise ascension, yet the sources of performance gains vary across paradigms.

It should be noted that the accuracies summarized in Tables 2–6 are reported results from the original studies rather than reproduced under a unified evaluation protocol; therefore, the following discussion emphasizes paradigm-level performance tendencies rather than strict numerical ranking.

Statistical and adversarial alignment paradigms represented the early mainstream approaches. Their limitation lies in the over-pursuit of mandatory alignment of marginal distributions, which often leads to “Semantic Misalignment”—sacrificing fine-grained class separability to minimize domain distance.

Topological and structural modeling and advanced representation learning paradigms have effectively broken through the bottleneck of single distribution alignment by introducing graph structural constraints and contrastive learning mechanisms. Notably, methods like STFCGAT (Li Z. et al., 2023) demonstrate that preserving subject-specific brain network topology is crucial for maintaining semantic integrity.

Multimodal fusion paradigms currently represent the “performance ceiling.” For instance, CFDA-CSF (Jimnez-Guarneros and Fuentes-Pineda, 2024) achieved an accuracy of 91.57 on SEED-V. This indicates that introducing heterogeneous information, such as eye movements or fNIRS, provides orthogonal feature supplementation, significantly reducing the uncertainty caused by EEG non-stationarity. However, this performance boost comes at the cost of increased hardware deployment difficulty.

### 5.2 Data dependency and labeling requirements

The core barrier to constructing a Zero-Calibration BCI lies in the degree of dependency on target domain data.

- High dependency: Traditional distribution alignment and topological modeling mostly belong to Transductive Transfer Learning. They typically assume access to unlabeled target domain data during the training phase. This limits the model's applicability in “plug-and-play” scenarios.
- Medium dependency: Generative modeling paradigms, such as CS-GAN (Chang et al., 2024) and TRANSIT-EEG (Ahuja and Sethia, 2025), alleviate the need for large-scale real data through data synthesis. However, their training processes still require covering sufficiently diverse source samples to learn a broad distribution of styles.
- Low dependency: Advanced representation learning paradigms show immense potential for reducing labeling dependency. For example, SASD-MCL (Zhang et al., 2025), utilizing self-supervised or semi-supervised strategies, can extract universal representations from unlabeled data. The future trend is evolving toward Source-Free Domain Adaptation (SFDA), where the model adapts to new users without backtracking to source domain data, offering decisive advantages for privacy protection and storage-constrained edge devices.

### 5.3 Physiological interpretability

Interpretability is critical for neuroscience research and clinical applications. Topological and structural modeling paradigms possess the strongest interpretability. By visualizing the adjacency matrices of dynamic graphs [e.g., DSDirGCN-AM (Shen et al., 2025)], researchers can directly observe asymmetric information flow between brain regions (such as the frontal and temporal lobes) during emotional processing, which aligns highly with findings in cognitive neuroscience.

The interpretability of distribution alignment and advanced representation learning paradigms is relatively weaker. Although some methods introduce attention mechanisms, adversarial or contrastive operations performed in deep feature spaces often remain “black boxes” that are difficult to map intuitively back to specific neurophysiological activities.

Generative models offer a new perspective by providing a “common-private” mode separation via style transfer, helping to understand which EEG patterns are subject-specific and which are shared across emotions.

### 5.4 Computational complexity and real-time performance

Given these clear differences in computational complexity, practical deployability becomes particularly important in wearable and portable EEG scenarios, where cross-subject emotion recognition models must operate under stricter constraints in terms of electrode number, memory footprint, inference latency, and power consumption. Recent studies have therefore shifted attention from purely accuracy-oriented model design to deployment-aware optimization. For example, Wang et al. (2023) proposed a lightweight domain adversarial neural network based on knowledge distillation, in which a high-capacity teacher model transfers temporal-spatial representation knowledge to a compact student network for cross-subject EEG emotion recognition. Liu F. et al. (2024) further investigated few-channel EEG emotion recognition by integrating deep feature aggregation and transfer learning, showing that knowledge learned from full-channel EEG can be used to compensate for the information loss caused by reduced electrode settings. In addition, Moontaha et al. (2023) developed an online learning pipeline for wearable EEG-based emotion classification, highlighting the importance of real-time model updating in practical wearable environments. More recently, Wen et al. (2026) explored portable EEG-based emotion recognition with reduced channel requirements, further demonstrating the practical demand for lightweight and channel-efficient EEG systems. These studies suggest that computational complexity should not be evaluated solely by model architecture, but also by deployment-oriented factors such as channel dependency, parameter size, inference latency, online adaptability, and hardware feasibility.

In terms of computational complexity, different methods exhibit a clear gradient:

- Lightweight: Statistical distribution alignment methods (e.g., CORAL, MMD) rely primarily on matrix operations. They have the lowest computational overhead and are suitable for fast inference on wearable chips following offline training.
- Medium-weight: Topological modeling methods require real-time construction of graph structures and calculation of graph convolutions. Their complexity grows non-linearly with the number of nodes (electrodes), posing challenges for latency in online processing.
- Heavyweight: Generative modeling and multimodal Transformer methods are the most computationally intensive. They not only involve massive parameters but may also require iterative sampling during inference. Consequently, they are difficult to deploy directly on low-power BCI headsets and typically rely on cloud computing or edge acceleration.

### 5.5 Summary

In conclusion, the core challenge of cross-subject emotion recognition lies in managing the trade-off between alignment intensity and semantic integrity.

The reported results summarized in Tables 2–6 provide a quantitative reference for this tendency. Specifically, statistical and adversarial distribution alignment methods report a broad accuracy range of approximately 52.12%–94.81% across different datasets and evaluation settings, indicating that although these methods are computationally efficient and can perform well on specific benchmarks, they remain sensitive to inter-subject and cross-dataset distribution shifts. Topological and structural modeling methods achieve approximately 65.28%–97.03%, showing a more favorable balance between accuracy and robustness by preserving brain-region relationships and spatiotemporal dependencies. Advanced representation learning methods exhibit the widest range, from approximately 58.10% to over 99%, suggesting strong potential for subject-invariant representation extraction but also high sensitivity to model architecture, training strategy, and evaluation protocol. Generative modeling and style reconstruction methods fall into a relatively concentrated moderate-to-high range of approximately 71.33%–92.83%, reflecting their value in alleviating data scarcity and subject-style variability. Multimodal complementary fusion methods report approximately 64.40% to over 98%, usually providing the highest performance ceiling, but this gain is accompanied by additional sensors, synchronization requirements, and increased system complexity.

Therefore, the comparison across paradigms should not be understood as a simple accuracy ranking, but as a multidimensional trade-off among performance, data dependency, physiological interpretability, and computational complexity. Early distribution alignment paradigms tended to forcefully pull distributions closer, often destroying the internal manifold structure of the data and leading to negative transfer. Topological modeling attempted to maintain semantics by preserving structure but often struggled in the face of drastic distribution drifts. Generative paradigms and advanced representation learning attempt to break this zero-sum game at a higher level of abstraction through strategies of “disentangle-then-align” or “reconstruction-generation.”

The ideal model of the future should be lightweight (for deployment), structure-aware (leveraging brain mechanisms), and capable of adaptive generation (handling unknown new users). This points the way from simple domain adaptation toward the integration of Domain Generalization and Causal Representation.

## 6 Discussion

Although cross-subject EEG emotion recognition has made significant progress driven by deep learning and transfer learning, establishing a rich methodological system ranging from statistical alignment to multimodal fusion, a significant gap remains between existing theoretical assumptions and engineering implementation during the transition to real-world Brain-Computer Interface (BCI) applications. Based on the technical limitations of the five paradigms analyzed above, this section analyzes the substantive challenges currently facing the field, with particular emphasis on semantic negative transfer, over-alignment, privacy-preserving adaptation, generative reliability, multimodal deployment cost, and the transition from correlation fitting to causal representation.

### 6.1 Risk of semantic negative transfer and over-alignment induced by mandatory distribution alignment

In physiological signal processing, existing distribution alignment methods typically rely on the assumption that the source and target domains share transferable feature distributions. However, due to differences in individual brain structures, skull conductivity, baseline neural activity, cognitive habits, and emotional appraisal patterns, the same emotional response may present substantially different EEG manifestations across subjects. This indicates not only distribution shift but also potential concept drift. Therefore, forcibly aligning data from different individuals into a unified distribution may achieve domain invariance mathematically, but may also cause the model to learn incorrect emotional semantics.

More specifically, semantic negative transfer may arise when non-transferable subject-specific patterns are mistakenly treated as emotion-related representations. In multi-source scenarios, physiologically distant source subjects may mislead the decision boundary if all source domains are treated equally. In class-conditional alignment, unreliable pseudo-labels may further pull samples from different emotional categories closer, thereby amplifying class confusion. This problem is closely related to over-alignment: excessive minimization of global domain discrepancies may collapse the local manifold structure of EEG data and weaken inter-class separability. In other words, a highly domain-invariant representation is not necessarily emotion-discriminative.

Future research should therefore shift from mandatory global alignment to selective and structure-preserving alignment. Models should estimate the transferability of source subjects or samples, reduce the influence of poorly transferable domains, and perform alignment at the class-conditional or sub-domain level. More importantly, neurophysiologically meaningful structures, such as functional connectivity patterns and frontal–temporal interactions, should be preserved during adaptation. The key issue is not only whether alignment should be performed, but what should be aligned, to what extent it should be aligned, and which subject-specific structures should be retained rather than eliminated.

### 6.2 Privacy compliance and cold-start dilemmas in transductive settings

Most current models (especially graph networks and representation learning approaches) require simultaneous access to the existing source database and the new user's target data during training, which is difficult to implement in practical applications. On one hand, there is the issue of privacy compliance: uploading existing user data to the cloud or transmitting it to a new user's terminal device violates data protection regulations. On the other hand, there is the cold-start problem: new users usually cannot afford the time to cooperate with extensive calibration data collection, leaving the model without sufficient information for adaptation. Future research should focus on Source-Free Domain Adaptation (SFDA) technology. This allows the model to self-adjust based on the new user's input features using pretrained model parameters without accessing the raw data of old users. This not only protects privacy but also realizes true “plug-and-play” capability.

### 6.3 Signal fidelity and computational cost of generative models

Although generative modeling offers new ideas for addressing EEG data scarcity, its engineering application faces challenges. EEG signals are inherently weak, unstable, and noisy; traditional generative models (such as GANs) easily synthesize samples with spurious high-frequency noise. Furthermore, emerging methods like diffusion models require multiple iterations for denoising, leading to high computational costs. Future research should not overly pursue the generation of raw signals but should turn to reconstructing emotional features in a low-dimensional latent space. By combining disentanglement ideas, models can generate only the compressed emotional features stripped of noise. This ensures the validity of generated data while significantly reducing the computational load, making it suitable for portable devices.

### 6.4 Trade-off between accuracy and hardware dependency in multimodal fusion

Multimodal fusion methods effectively improve the accuracy of EEG emotion recognition by combining signals like eye movements and facial expressions, but this often requires additional sensors, resulting in bulky equipment, increased power consumption, and inconvenience in wearing. Moreover, subtle synchronization biases between multimodal signals can easily affect model performance. In practical applications, multimodal fusion also faces the challenges of missing modalities and modality mismatch between training and testing. Most existing methods assume that all modalities are simultaneously available during both training and inference, whereas auxiliary signals may be absent or degraded due to sensor detachment, facial occlusion, illumination variation, eye-tracker calibration drift, or unstable acquisition environments. In addition, eye movements and facial expressions are still behavioral signals and may be affected by intentional masking, voluntary control, social display rules, or individual expressive habits. Therefore, they are not always more objective or reliable than EEG. Another issue is that different devices usually have inconsistent sampling rates, temporal resolutions, and system latencies, which may further introduce cross-modal temporal mismatch if simple feature concatenation or coarse synchronization is adopted. The future trend will increasingly leverage learning using privileged information and knowledge distillation. This involves using multimodal data during the training phase to teach the EEG model how to utilize auxiliary information, while relying solely on a single EEG modality during actual usage. This approach retains high accuracy without requiring users to wear extra hardware.

This direction is also consistent with recent lightweight and wearable EEG emotion recognition studies. In real-world wearable scenarios, users usually cannot tolerate dense electrode montages, multiple auxiliary sensors, or cloud-dependent inference. Therefore, practical systems should move toward few-channel acquisition, compact model design, and on-device adaptation. From this perspective, the future of multimodal fusion may not lie in adding more sensors during deployment, but in transferring multimodal knowledge into a lightweight single-EEG model during training. This would allow multimodal information to improve model robustness while maintaining the hardware simplicity required for wearable affective BCI systems.

### 6.5 Paradigm shift from correlation fitting to causal representation

Essentially, all current mainstream methods seek statistical correlations within the data. However, EEG signals are mixed with numerous features that are strongly correlated with individual identity (e.g., baseline fluctuations caused by skull differences) but irrelevant to emotion. This makes models prone to classifying based on identity features rather than emotion-related neural patterns, leading to a sharp performance drop on unfamiliar subjects. Introducing causal inference will be a key direction for future research. By explicitly defining individual differences as confounding factors, models can focus on learning stable, causal emotional representations. In this context, future studies should not only suppress identity-related nuisance variables, but also attempt to estimate emotion-related effective connectivity or causal interaction graphs among brain regions. Such causality-aware representations may provide a more robust foundation for cross-subject generalization than purely correlation-based feature alignment.

Recent studies on dementia-related causality analysis further support this transition from correlation fitting to causal representation. Zuo et al. (2025) proposed a brain imaging-to-graph generation framework based on adversarial hierarchical diffusion models for MCI causality analysis. By mapping fMRI signals into effective connectivity graphs, this framework attempts to infer causal interaction patterns among brain regions rather than merely fitting statistical correlations. Although the task scenario differs from EEG emotion recognition, it provides an important methodological implication: future affective BCI models may benefit from generating or estimating emotion-related effective connectivity graphs, thereby identifying stable causal neural pathways underlying emotional responses. This direction is particularly valuable for cross-subject generalization, because causal brain connectivity patterns are expected to be more stable than superficial subject-specific signal distributions.

## 7 Conclusion

Cross-subject EEG emotion recognition is the critical technology for breaking the barrier of Brain-Computer Interfaces moving from the laboratory to real-world applications. Addressing the challenges of the inherent non-stationarity of EEG signals and significant inter-subject variability, this paper transcends previous limitations of categorization based solely on network architectures and proposes a novel taxonomic system based on the “Generalization Hypothesis.” By reconstructing existing literature into five major paradigms—statistical and adversarial distribution alignment, topological and structural modeling, advanced representation learning, generative modeling and style reconstruction, and multimodal complementary fusion—this paper systematically outlines the methodological evolution and theoretical core of the field.

A comprehensive analysis indicates that the research focus in this field is shifting from emphasizing mandatory statistical alignment toward structural and generative modeling, which demonstrate greater potential in maintaining emotional semantic integrity and addressing data distribution discrepancies. However, current high-performance models generally suffer from dependency on target domain data, high computational overhead, dense electrode requirements, or complex hardware configurations, which remains a significant gap compared with the practical demands for lightweight, wearable, plug-and-play, and privacy-preserving systems. Recent lightweight and wearable EEG studies further highlight that practical cross-subject emotion recognition should move beyond offline accuracy optimization and incorporate deployment-oriented criteria, including channel efficiency, real-time inference capability, energy consumption, and hardware feasibility.

The breakthrough for future research should not only lie in fundamental paradigm innovation, but also in deployment-oriented and experimentally verifiable system design. From a methodological perspective, integrating Source-Free Domain Adaptation can help address data privacy and cold-start dilemmas; introducing Causal Representation Learning can strip away spurious correlations related to identity; and combining privileged information distillation can retain multimodal informational advantages under unimodal deployment. From an engineering perspective, future models should be designed under real-world constraints, including limited calibration time, unstable electrode contact, cross-session signal drift, motion artifacts, low-power wearable hardware, and strict privacy requirements.

More specifically, future studies should formulate these directions as targeted and testable research problems for practical deployment. First, source-free and online self-adaptation should test the hypothesis that new users can obtain reliable recognition performance with minimal or no calibration under strict leave-one-subject-out, cross-session, and streaming protocols. Potential risks such as pseudo-label confirmation bias, catastrophic adaptation to noisy samples, and unstable electrode contact should also be considered. Second, causal representation learning should test whether emotion-related neural patterns can be separated from subject-specific confounders without losing discriminative semantics, using identity-discriminability tests, cross-dataset validation, robustness to artificial distribution shifts, and interpretability analysis. Third, privileged-information distillation should test whether a “training-rich, deployment-light” strategy can preserve multimodal advantages under EEG-only inference, missing-modality settings, asynchronous sampling conditions, and wearable-device constraints. Finally, future evaluation should move beyond accuracy-oriented comparisons and incorporate deployment metrics such as latency, memory consumption, energy cost, calibration duration, cross-day robustness, and failure rate under noisy environments. Privacy-preserving learning frameworks, such as source-free adaptation, federated optimization, and on-device updating, should also be further explored to ensure safe use in personal healthcare and human-computer interaction scenarios.

Only by making substantial progress on both foundational learning paradigms and verifiable deployment-oriented system optimization can affective Brain-Computer Interfaces build truly robust, interpretable, lightweight, and user-friendly systems, thereby reliably serving a wide range of mental health monitoring and human-computer interaction applications.
